# Analysis of Pericardial Effusion from Idiopathic Pericarditis Patients by Two-Dimensional Gel Electrophoresis

**DOI:** 10.1155/2014/942718

**Published:** 2014-04-02

**Authors:** Sadan Yavuz, Murat Kasap, Gurler Akpinar, Ersan Ozbudak, Dilek Ural, Turan Berki

**Affiliations:** ^1^Department of Cardiovascular Surgery, Kocaeli University Medical School, Kocaeli, Turkey; ^2^Department of Medical Biology, DEKART Proteomics Laboratory, Kocaeli University Medical School, Umuttepe, 41380 Kocaeli, Turkey; ^3^DEKART Proteomics Laboratory, Kocaeli University Medical School, Kocaeli, Turkey; ^4^Department of Cardiology, Kocaeli University Medical School, Kocaeli, Turkey

## Abstract

Pericardial fluid (PF) is often considered to be reflection of the serum by which information regarding the physiological status of the heart can be obtained. Some local and systemic disorders may perturb the balance between synthesis and discharge of PF and may cause its aberrant accumulation in the pericardial cavity as pericardial effusion (PE). PE may then lead to an increased intrapericardial pressure from which the heart function is undesirably affected. For some cases, the causes for the perturbance of fluid balance are well understood, but in some other cases, they are not apparent. It may, thus, be helpful to understand the molecular mechanisms behind this troublesome condition to elucidate a clinical approach for therapeutic uses. In this study, protein profiles of PEs from idiopathic pericarditis patients were analyzed. Control samples from patients undergoing elective cardiac surgery (ECS) were included for comparison. In addition to high abundant serum-originated proteins that may not hold significance for understanding the molecular mechanisms behind this disease, omentin-1 was identified and its level was higher for more than two-fold in PE of IP patients. Increased levels of omentin-1 in PE may open a way for understanding the molecular mechanisms behind idiopathic pericarditis (IP).

## 1. Introduction

The space between the parietal and visceral layers of the pericardium contains small amount of fluid called PF. PF has a discernible lubricant function [1, 2]. The composition of normal PF can be described as an ultrafiltrate of plasma except with low protein content [3–5]. Some systemic and local disorders such as coronary artery diseases, malignant diseases, connective tissue disorders, idiopathic causes, inflammation, tumors, or hemorrhage may disturb the balance between formation and removal of PF and cause its accumulation as PE [5, 6].

The causes of pathological PE are not always clear and the etiology is unknown in more than 50% of the cases [7–9]. A systematic approach for diagnostic testing based on standardized practice guidelines has been proposed [10]. A diagnostic pericardiocentesis and/or pericardial biopsy are/is recommended for large/recurrent effusions if conventional tests remain inconclusive. Unfortunately, analysis of the biochemical and cell-count composition of the pericardial fluid is generally not helpful for the diagnosis of most pericardial effusions [11]. Therefore, a large proportion of the cases are labeled as idiopathic pericarditis (mean: 26.1%), followed by neoplastic diseases (mean: 25.6%) and iatrogenic pericarditis (mean: 16.3%) [9].

By using biochemical approaches, the presence of putative biomarkers like CRP was proposed in PE of pericarditis patients [8, 12, 13]. However, those biomarkers did not find place in clinical practice. Proteomic approaches may help to identify incipient biomarkers to fulfill the needs in cardiovascular diseases including pericarditis [14]. However, until recently an extensive study examining the potential utilization of PF as a source of biomarkers was missing. Fortunately, recently published study reported an extensive list of low abundant proteins from PF and highlighted that, as a biochemical window of heart, PF proteome can be a good material for cardiovascular research [15]. In this study, we used two-dimensional gel electrophoresis (2D) to examine the protein profile of PE from IRP patients. The results showed that, unlike the control samples from ECS patients, omentin-1 can readily be detectable in 2D gels prepared from PE samples making it a putative marker for the disease.

## 2. Methods

The study was approved by the institutional review board and informed consents were obtained from all patients.

### 2.1. Sample Collection 

A subxiphoid vertical incision was made under general anesthesia and pericardial cavity was entered. After opening a pericardial window, a pericardial biopsy was taken and drainage was performed. The pericardial fluid samples were subjected to biochemical, microbiological, and pathological examinations. Thoracic tomography and ultrasonography were performed to all patients for tumor detection. The study group was composed of seven IRP patients for whom no diagnosis was possible to explain the presence of PE. Blood-free PE samples were collected in sterile tubes without anticoagulant. Similarly, PF samples from ECS patients were collected to form a control group. After centrifugation at 3000 ×g for 10 min at 4°C, the supernatants were collected and aliquoted into Lo-Bind storage tubes (Eppendorf Inc., USA) and stored at −80°C until use. The protein concentrations were measured with RC-DC protein assay (Bio-Rad, USA).

### 2.2. MicroRotofor Fractionation

One mL of each sample was desalted through a 10 DG column (Bio-Rad, USA) and buffer exchange was performed with 10 mM Tris.Cl, pH 6.8. After combining protein containing fractions that were eluted from 10 DG column, three mL of the combined fractions was mixed with 40% ampholyte (pH 3–10) to obtain 2% final ampholyte concentration. The sample was then loaded to a MicroRotofor unit (Bio-Rad, USA) and focused for 3 hr at 1 W. At the end of the focusing period, ten fractions from each sample were collected and 5 µL of each fraction was subjected to SDS-PAGE for analysis of fractionation efficiency. To remove the excess ampholyte that originated from MicroRotofor fractionation, the fractions were dialyzed against 100-fold diluted 2D sample buffer by using a Slide-A-Lyzer dialysis unit with a MW cut-off limit of 2000 (Pierce, USA) and carefully recovered without a significant protein loss.

### 2.3. Two-Dimensional Gel Electrophoresis (2DE)

Protein fraction number four of each sample obtained from MicroRotofor was subjected to 2DE analysis. Eighty µg of protein was actively (50 V) loaded to IPG strips (11 cm, pH 5–8, Bio-Rad) at 20°C for 16 hr and then run through a stepwise incremental voltage program (250 V for 20 min (linear), 4000 V for 2 hr (linear), and 10000 V/hr (rapid)) by using Protean IEF system (Bio-Rad, USA). The strips were then subjected to a two-step equilibration in equilibration buffers containing 6 M urea, 2% SDS, 0.375 M Tris.Cl pH 8.8, 20% glycerol and 2% DTT for the first step and 6 M urea, 2% SDS, 0.375 M Tris.Cl pH 8.8, 20% glycerol and 2.5% iodoacetamide for the second step. The strips were then transferred onto the second-dimension SDS-PAGE equipment and proteins were separated on 12% polyacrylamide gels. Protein spots were visualized by using SyproRuby fluorescent stain.

### 2.4. Image Acquisition and Analysis

Gel images were taken with an imaging system (VersaDoc4000MP, Bio-Rad, USA) and analyzed by using PDQuest Advanced 2D-image analysis software (Bio-Rad, USA). The quantity of each spot was normalized using local regression model. Based on average spot volume ratio, spots whose relative expression levels were changed at least 2-fold (increase or decrease) among the compared groups were considered to be significant. Statistical significance was assessed by using student's *t*-test (*P* < 0.01). Protein spots that displayed statistically significant regulation were cut by using automated EXQuest Spot Cutter (Bio-Rad) and deposited in a 96-well plate for in gel-tryptic digestion.

### 2.5. Tryptic In-Gel Digestion and MALDI-TOF/TOF Analysis

MALDI-TOF MS and TOF/TOF tandem MS/MS were performed by Applied Biomics (http://www.appliedbiomics.com/index.html; Hayward, CA, USA) using an AB SCIEX TOF/TOF 5800 System (AB SCIEX). The resulting peptide mass and the associated fragmentation spectra were submitted to GPS Explorer workstation equipped with MASCOT search engine (Matrix Science Inc.) to search the National Center for Biotechnology. Additional information for the MS/MS database search parameters and protein identification can be found in Supplementary Tables 1, 2, and 3, respectively (see Supplementary Materials available online at http://dx.doi.org/10.1155/2014/942718).

### 2.6. Western Blot Analysis

Equal volume of PE/PF from fraction four of each fractioned sample was mixed to form protein pools of study and control groups, respectively. After SDS-PAGE electrophoresis, proteins were transferred to a nitrocellulose membrane from an SDS-PAGE gel using a semidry transfer apparatus following the instructions provided by the manufacturer (TurboBlot, Bio-Rad, USA). The membrane was then probed with an anti-omentin-1 monoclonal antibody (Clontech, USA) using the chemiluminescent detection system (GE Healthcare, USA). The images were recorded with VersaDoc MP4000 (Bio-Rad, USA) and a set of prestained protein markers (Fermentas, USA) was used to assess the size of the signal (~40 kD) generated in western blots. For the purpose of spot analysis, ImageJ, freely available software, was used. The integrated density of each protein band was measured by outlining them and using the analyze/measure command.

## 3. Results 

Clinical features of seven patients and five controls were presented at Table 1. There was no significant difference among the IP and ECS patients in their demographic properties and standard serum biochemical test results. All of the studied samples were transudative. There was no history of trauma and hemorrhage in any of the subjects. Microbiological examination of effusions and pericardial tissue biopsies indicated no definite bacterial or fungal infections. Bacterial staining tests for acid-alcohol fast bacteria and mycobacteria were negative, and serum adenosine deaminase (ADA) screening remained negative for tuberculosis. Serologic screening for autoantibodies indicated no abnormality as well. Cytological and pathological examination of tissue samples revealed the absence of malign cells, and this finding was also supported by the low levels of serum tumor markers (Ca-125, Ca 15-3, Ca 19-9, CEA and AFP) (data not shown).

**Figure 1:**
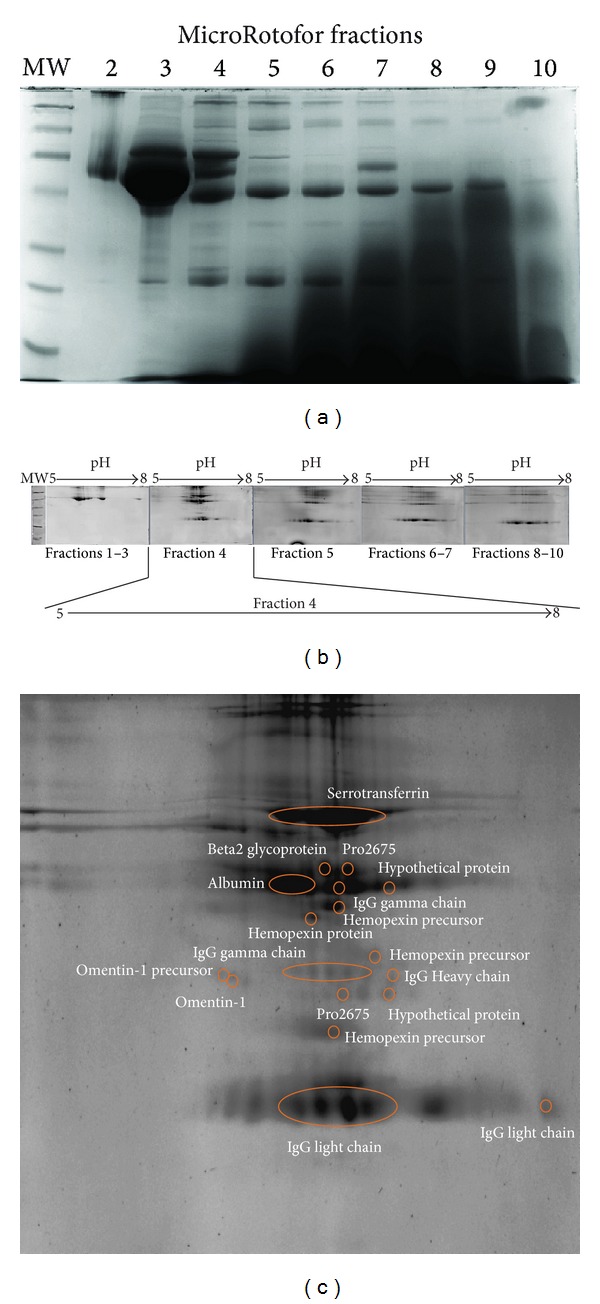
(a) SDS-PAGE analysis of MicroRotofor fractions of a PE after 3 hr. of focusing at 1 W. Notice that majority of the albumin was collected in fraction 3. The shadowy darkness at an increasing pattern was due to ampholyte (pH 3–10) that was added to the protein mixture at a concentration of 2% before focusing. (b) 2D analysis of MicroRotofor fractions. Fraction 4 was the only fraction that contained detectable level of omentin-1. (c) Positions of the protein spots that were identified in fraction 4. Despite the extensive prefractionation, high abundant proteins were still dominating the gel.

When samples were subjected to SDS-PAGE without prefractionation, high abundant proteins were apparent and needed to be reduced to enrich low abundant proteins. A Isoelectric Point (pI)-based fractionation approach was used to allow detection of low abundant proteins. SDS-PAGE analysis of each fraction revealed that fractionation enriched some of the minor proteins by placing majority of the albumin into a single fraction (Figure 1(a)). 2D analysis of each fraction confirmed this finding. Fraction number four contained protein spots that were otherwise not detectable on 2D gels prepared from unfractionated samples. (Figures 1(b) and 1(c)). MALDI-TOF/TOF analysis of some of the selected spots indicated the presence of abundant proteins such as albumin or albumin in a complex with myristic and triiodobenzoic acids (Pro2675), immunoglobulin, and hemopexin (Figure 1(c), Supplementary Table 2). These spots matched with the plasma 2DE-map [16].

**Figure 2:**
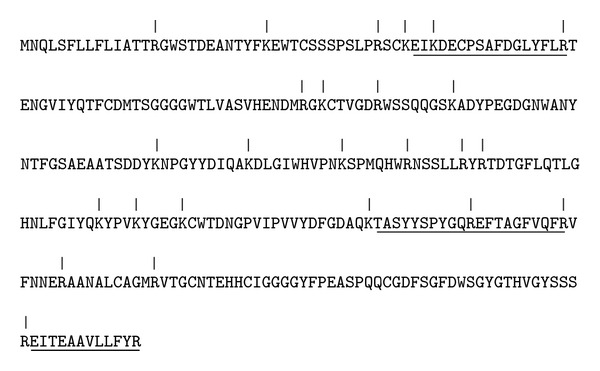
Complete amino acid sequence of omentin-1. Vertical lines represent possible tryptic digestion points and underlined peptides are the ones detected by MALDI-TOF/TOF analysis.

**Figure 3:**
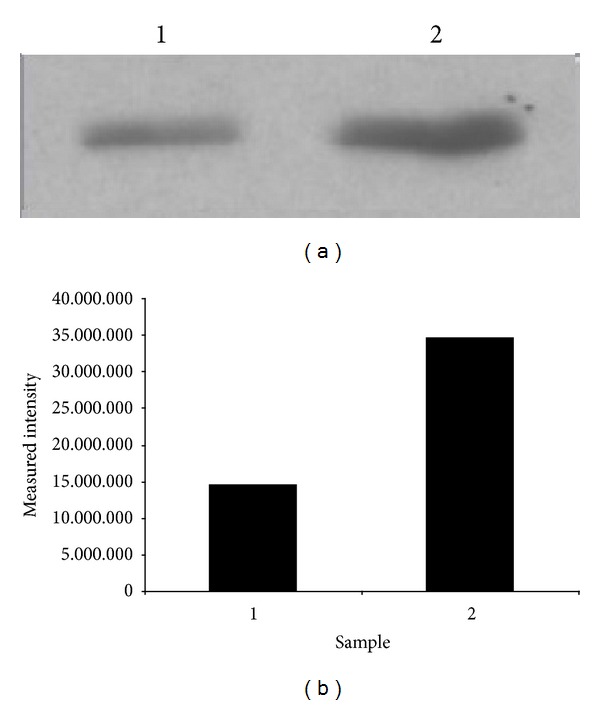
(a) Western blot analysis of the pooled PF samples from ECS patients and PE samples from IP patients. The 4th fraction after MicroRotofor fractionation was pooled and used for analysis. (b) Graphical presentation of the band intensities measured with ImageJ.

Among the identified spots, peptides belonging to omentin-1 (alternative name: intelectin-1) were readily detectable in PE samples (Table 2, Supplementary Table 3). MALDI-TOF/TOF analysis identified four peptides out of 26 possible tryptic peptides which accounted for the recovery of 16% of whole omentin-1 sequence with high confidence interval (Figure 2). When WB analysis was performed from protein pools of the study and control groups, omentin-1 was found to be present more in the pooled sample from the study group. Measurement of band intensities revealed more than 2-fold increase in omentin-1 levels (Figure 3).

## 4. Discussion 

As a biochemical window of heart, PF may hold the potential as a biomarker to assist in diagnosis of various heart diseases. Because biomarkers are mostly proteins, studying protein profile of PF may hold great importance. However, there have been limited efforts to perform an in-depth analysis of the PF proteome. This may be due to the fact that PF is a hard to reach material to reach and its collection requires invasive procedures. In a previous study, Liu et al. (2008) described tuberculosis related proteins in PE samples obtained from tuberculosis patients with heart failure and identified a number of differentially expressed tuberculosis-related proteins [17]. However, their study failed to describe a cardiac related protein as a biomarker. In a recent study, Xiang et al. (2013) described proteomic profiling of PF and identified over 1000 nonredundant proteins to generate the first comprehensive PF proteome (Xiang et al., 2013). However, their study excluded the patients with evidence or history of cardiac or pericardial diseases and thus is only descriptive in nature. In this study, we used 2DE approach to examine protein profiles of PE samples from IP patients and compared them with protein profiles of PF samples of patients undergoing ECS hoping to find a potential biomarker for differentiation of IP.

The only protein that created interest in terms of its elevated presence in the IP patients was omentin-1 (NCBI accession # 119573073). Omentin-1 is a relatively recently identified novel adipocytokine whose involvement in obesity, insulin resistance, and diabetes is recognized [18–21]. In addition, omentin-1 levels are altered in chronic inflammatory conditions particularly in autoimmune diseases [22]. In fact, omentin-1 was proposed to be a potential biomarker in synovial fluid for reflecting the degenerative process in osteoarthritis [23]. The involvement of omentin-1 in cancer was also proposed based on the finding that omentin-1 gene overexpression was 139-fold higher in malignant pleural mesothelioma cells [24]. Circulating omentin-1 levels was also proposed to be an independent marker for arterial stiffing in patients with type-2 diabetes [25, 26]. In some studies, omentin-1 levels were measured and associated with cardiovascular diseases [27, 28].

Although omentin-1 is a main indicator of inflammation, these and similar studies clearly demonstrated the pleiotropic nature of omentin-1 which appears to have a role in regulating various metabolic events in our bodies [29]. Therefore, a detailed understanding of themolecular mechanisms by which these regulations occur is needed. In this study, we reported the elevated levels of omentin-1 in PE of IP patients and proposed that omentin-1 might be an indicator of the disturbed pericardial balance.

The etiology and pathogenesis of IP remain controversial standing like a bridge that crosses infectious, autoimmune, and autoinflammatory pathways [29]. Microorganisms such as viruses, bacteria, and fungi can cause the pericardial infections. The most common viral pathogen is known to be coxsackie virus and echovirus. Other common agents are cytomegalovirus, herpes virus, and HIV [30, 31]. However, details of pericarditis caused by other infectious agents are not yet known. Therefore, various treatment strategies are employed. Nonsteroidal anti-inflammatory drugs must be used at recommended dosages to resolve the symptoms so that normalization of C-reactive protein and erythrocyte sedimentation rate are reached [31]. Corticosteroids should be used rarely, at low doses, with an extremely low tapering and with osteoporosis prevention [32]. Colchicine leads to a clinically important and statistically significant benefit, reducing recurrences. Surgical treatment of pericardial pathologies is reserved for symptomatic patients. The choice of procedure could be partial or complete pericardiectomy, pericardioplasty or actualincision. Longitudinal extension of the defect to relieve tension on critical structus performed [33].

## 5. Conclusions 

In conclusion, the value of pericardial fluid as a biomarker source for the detection of cardiovascular diseases cannot be underestimated. When the dynamic nature of pericardial fluid—its continuous reproduction and the drainage—is considered, its importance becomes more apparent in biomarker discovery research. In this study, the proteomes of PEs were examined to some extent.

## Supplementary Material

Supplementary Table 1: Information on MS/MS database search.
Supplementary Table 2: contains identification scores for the list of identified proteins.
Supplementary Table 3: contains peptide sequences that were detected by MALDI TOF TOF.

## Figures and Tables

**Table 1:** Demographic features and biochemical test results for the RIP and ECS patients.

Demographic features	Pericardial effusion							Elected cardiac surgery				
	1	2	3	4	5	6	7	1	2	3	4	5
Sex (male/female)	F	F	M	M	F	M	F	M	M	F	F	M
Age (year)	58	45	63	70	59	42	69	41	63	53	65	72
Hypertension (+/−)	**+**	**−**	**+**	**+**	**+**	**−**	**−**	**−**	**−**	**+**	**+**	**+**
Diabetes mellitus (+/−)	**−**	**−**	**−**	**−**	**−**	**−**	**−**	**−**	**−**	**+**	**−**	**+**
Myocardial infarction (+/−)	**−**	**−**	**−**	**−**	**−**	**−**	**−**	**−**	**+**	**+**	**−**	**−**
Ejection fraction (%)	66	60	65	68	88	67	56	45	40	30	60	78

Biochemical test results												
BUN (mg/dL)	13.1	10	15	9	14	18	13	13	17	20	16.5	21
Creatinine (mg/dL)	0.65	0.66	0.5	0.8	0.9	0.71	0.7	0.8	0.9	0.6	0.79	1.02
GOT (U/L)	18	11	28	16	17	25	60	18	53	23	28	24
GPT (U/L)	16	15	18	11	12	38	14	22	37	21	18	18
Albumin (g/dL)	4.41	3.9	3.1	3.42	3.4	3.45	3.6	4.4	4	3.5	3.6	3.9
LDH (U/L)	216	215	251	205	230	169	334	237	210	183	178	192
TP (g/dL)	7.26	7.1	6.6	6.5	5.9	6.3	8.2	7.1	7.3	6.8	7	7.5
LDH (PE) (U/L)	95	130	141	138	167	102	147					
TP (PE) (g/dL)	2.2	5.1	3.6	3.4	2.8	3.2	3.6					
CRP (mg/L)	0.231	0.293	0.6	0.23	2.03	1.7	0.6	0.3	0.2	0.76	1.26	0.29
ADA (u/L)	9.20	22	15	24	38	18	23					
TSH (Uıu/mL)	0.994	0.665	1.203	1.27	0.22	1.69	4.4	0.8	0.768	0.63	0.71	1.11

**Table 2:** Tryptic peptides of omentin-1 identified by MALDI-TOF/TOF analysis.

Calculated mass	Observed mass	±Da	±ppm	Start sequence	End sequence	Sequence
2059.9792	2059.8721	−0.1071	−52	43	59	EIKDECPSAFDGLYFLR
1292.5906	1292.6897	0.0991	77	219	229	TASYYSPYGQR
1201.6	1201.6957	0.0957	80	230	239	EFTAGFVQFR
1424.7783	1424.8894	0.1111	78	302	313	EITEAAVLLFYR
