# Supplementary material for: Analysis of Pericardial Effusion from Idiopathic Pericarditis Patients by Two-Dimensional Gel Electrophoresis
Source: Biomed Res Int. 2014 Apr 2;2014:942718. doi: 10.1155/2014/942718 (PMC3996951; doi:10.1155/2014/942718)
Supplement: Supplementary file 1 — Supplementary Table 1: Information on MS/MS database search. Supplementary Table 2: contains identification scores for the list of identified proteins. Supplementary Table 3: contains peptide sequences that were detected by MALDI TOF TOF. [file 942718.f1.pdf]

Supplementary Table 1: Information on MS/MS database search

**Information on MS/MS database search:**	
a. Name of peaklist-generating software and release version (number or date)	GPS Explorer ™ v3.6
b. Parameters used – default vs altered	n/a
c. Name of the search engine and release version (number or date)	MASCOT 2.0 (Matrix Science)
Search Parameters:	
d. Enzyme specificity considered	Trypsin
e. # of missed cleavages permitted	1
f. Variable modifications (including residue specificity)	Carbamidomethylation of cysteine, oxidation of methionine/histidine/tryptophan, phosphorylation of serine/threonine/tyrosine, acetylation of lysine, and methylation of histidine/lysine/arginine
g. Mass tolerance for precursor ions	50 ppm
h. Mass tolerance for fragment ions	0.4 Da
i. Name of database searched and release version/date	nr NCBI or Swiss-Prot (automatically updated to most current version)
j. Species restriction and justification for searching only a subset of a database	*Homo sapiens*

l. Cut-off score/expectation value for accepting individual MS/MS spectra	S/N cutoff: 20
m. Justification of the threshold employed	noise is generally less than 5%
